# Supplementary material for: Oligomerization and Adjuvant Activity of Peptides Derived from the VirB4-like ATPase of Clostridioides difficile
Source: Biomolecules. 2023 Jun 18;13(6):1012. doi: 10.3390/biom13061012 (PMC10296573; doi:10.3390/biom13061012)
Supplement: Supplementary file 1 [file biomolecules-13-01012-s001.zip › Oligomerization_BelyiY_SuppleFigs_2023-05-20.pdf]

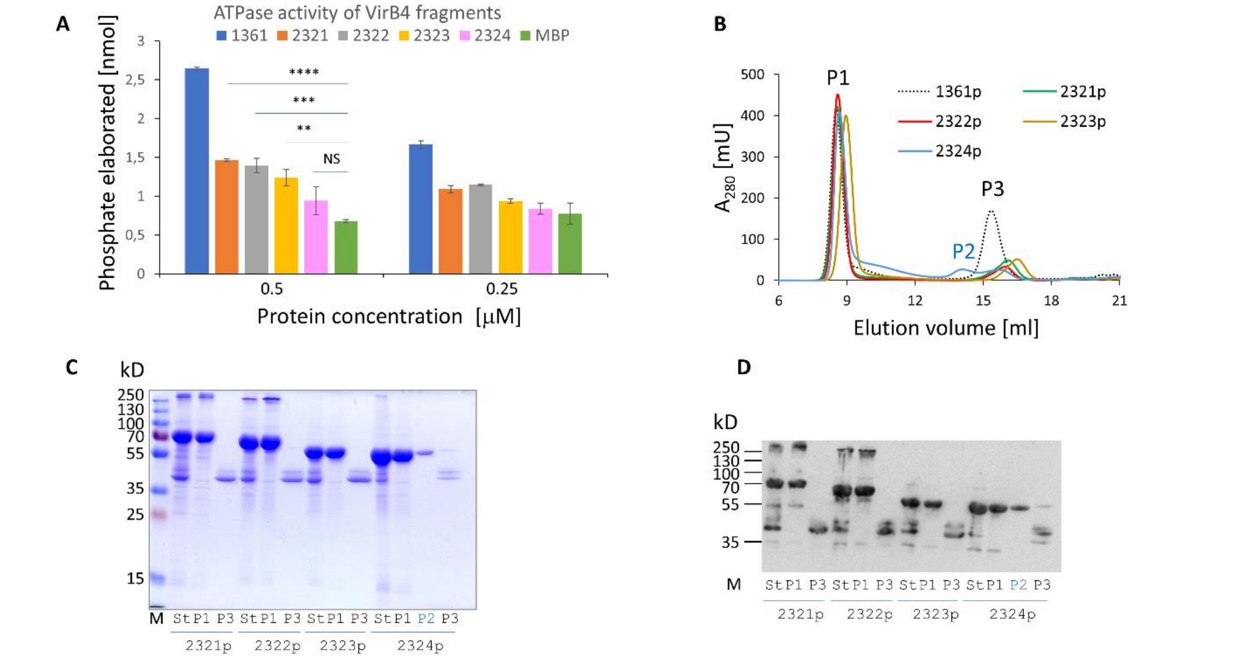

Figure S1. Biochemical properties of chimeric proteins containing large VirB4 fragments. **A**, ATPase activity of the VirB4 fragments fused to MBP. Data is presented as a mean and standard deviation. Difference between the groups is calculated according to Student t-test as not-significant (NS), or significant at P<0,001 (****), P<0,005 (***), P,0,01 (**). **B**, Superose 6 analytical chromatography of the chimeric proteins. Fractions from peaks (P1, P2, P3) are analyzed and shown in **C**. If the peak is seen with a single protein, it is marked by the corresponding line color. X-axis represents elution volume in ml, while Y-axis shows optical density of solution at 280 nm. **C**, SDS-PAGE analysis of fractions from the main peaks on panel B. St, starting material (proteins purified by the MBPtrap chromatography); M, molecular mass markers. **D**, Western blot analysis of fractions from the main peaks on panel B with the anti-MBP serum.

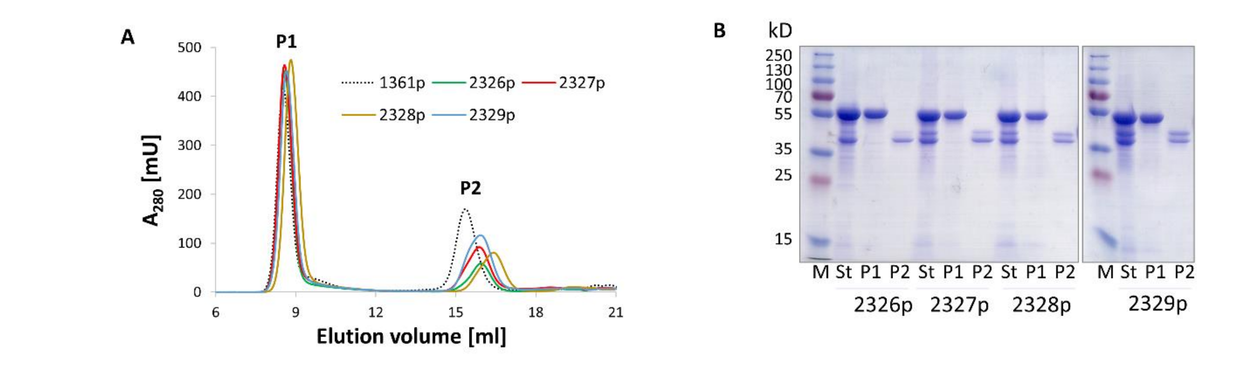

Figure S2. Biochemical analysis of chimeric proteins. **A**, analytical gel-chromatography on Superose 6. X-axis represents elution volume in ml, while Yaxis shows optical density of solution at 280 nm**. B**. SDS-PAGE analysis of the fractions from the main peaks on panel **A**. St, starting material (proteins purified by the MBPtrap chromatography); M, molecular mass markers.

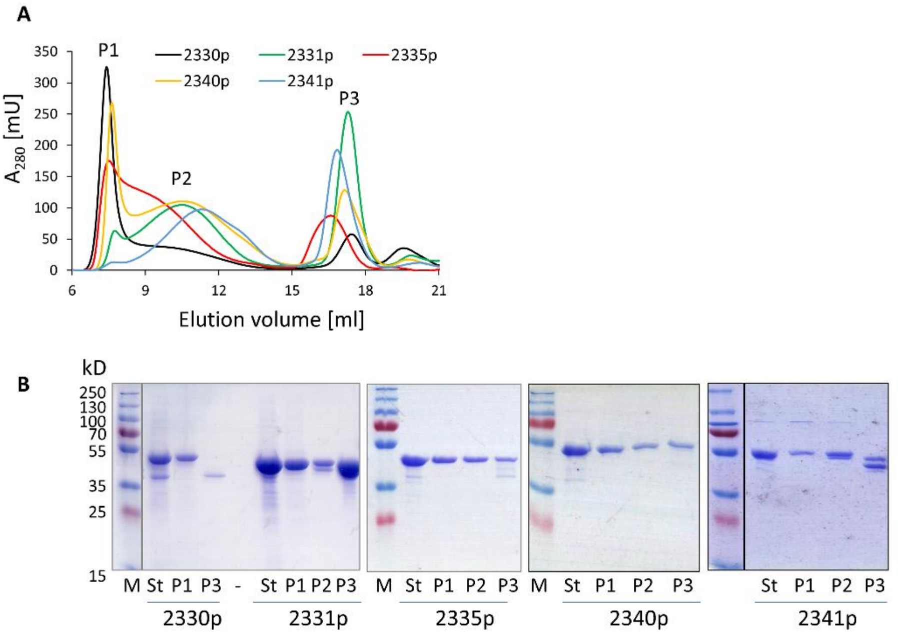

Figure S3. Biochemical analysis of chimeric proteins. **A**, analytical gel-chromatography on Superose 6. X-axis represents elution volume in ml, while Yaxis shows optical density of solution at 280 nm**. B**. SDS-PAGE analysis of the fractions from the main peaks on panel **A**. St, starting material (proteins purified by the MBPtrap chromatography); M, molecular mass markers.

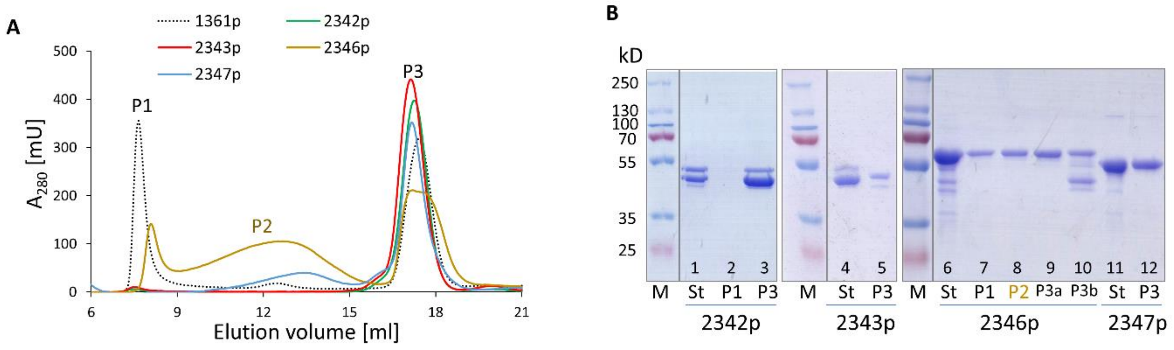

Figure S4. Biochemical analysis of chimeric proteins. **A**, analytical gel-chromatography on Superose 6. X-axis represents elution volume in ml, while Yaxis shows optical density of solution at 280 nm**. B**. SDS-PAGE analysis of the fractions from the main peaks on panel **A**. St, starting material (proteins purified by the MBPtrap chromatography); M, molecular mass markers.

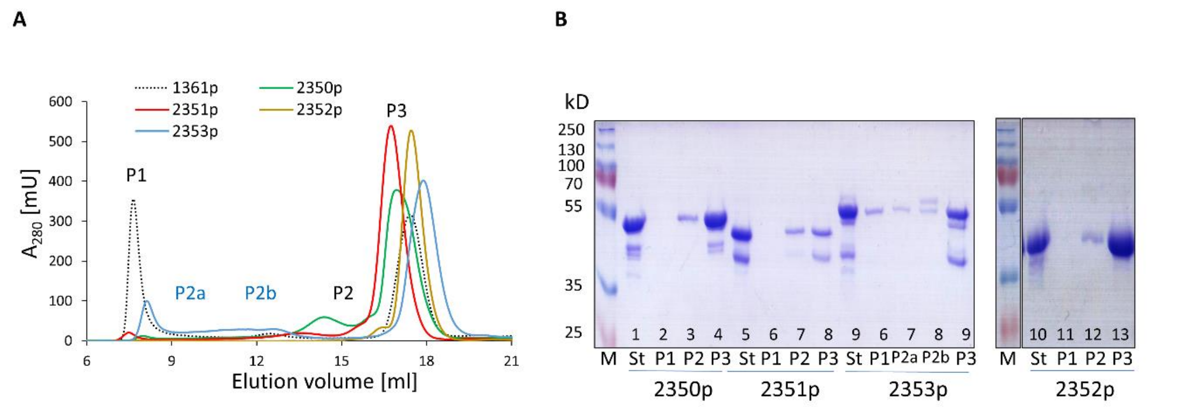

Figure S5. Biochemical analysis of chimeric proteins. **A**, analytical gel-chromatography on Superose 6. X-axis represents elution volume in ml, while Yaxis shows optical density of solution at 280 nm. **B**. SDS-PAGE analysis of the fractions from the main peaks on panel **A**. St, starting material (proteins purified by the MBPtrap chromatography); M, molecular mass markers.

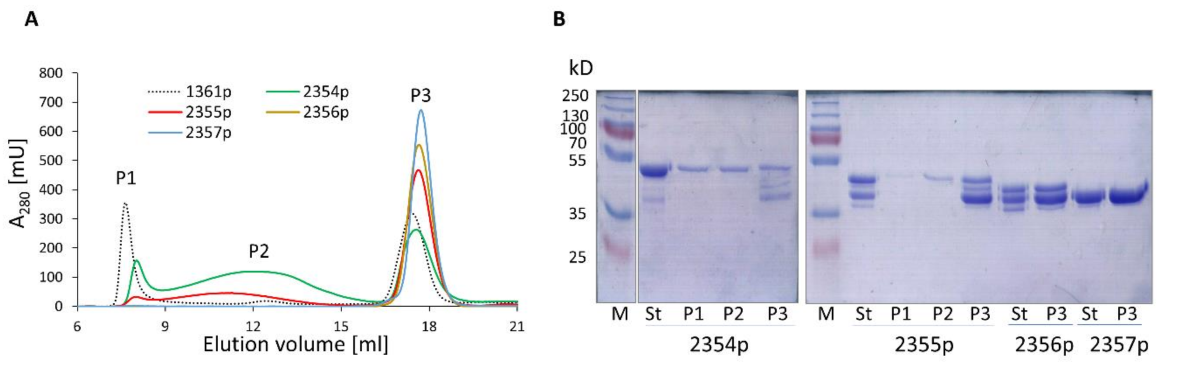

Figure S6. Biochemical analysis of chimeric proteins. **A**, analytical gel-chromatography on Superose 6. X-axis represents elution volume in ml, while Yaxis shows optical density of solution at 280 nm. **B**. SDS-PAGE analysis of the fractions from the main peaks on panel **A**. St, starting material (proteins purified by the MBPtrap chromatography); M, molecular mass markers.

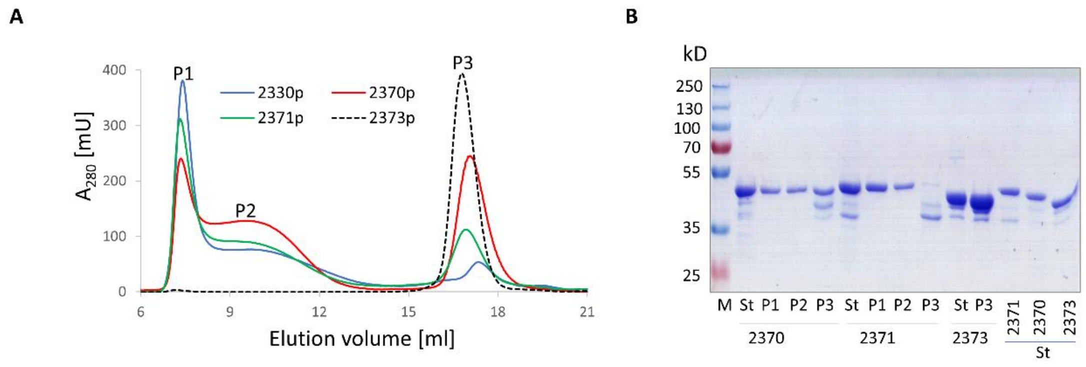

Figure S7. Biochemical analysis of chimeric proteins. **A**, analytical gel-chromatography on Superose 6. X-axis represents elution volume in ml, while Yaxis shows optical density of solution at 280 nm. **B**, SDS-PAGE analysis of the fractions from the main peaks on panel **A**. St, starting material (proteins purified by the MBPtrap chromatography); M, molecular mass markers.

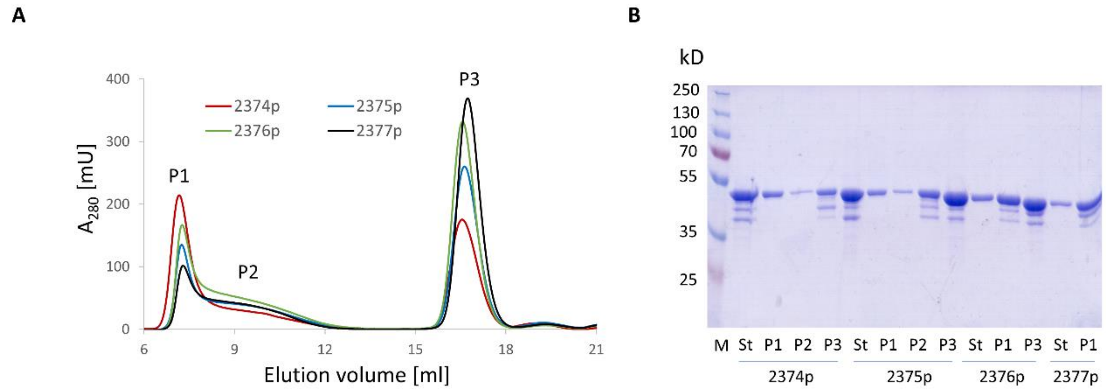

Figure S8. Biochemical analysis of chimeric proteins. **A**, analytical gel-chromatography on Superose 6. X-axis represents elution volume in ml, while Yaxis shows optical density of solution at 280 nm. **B**, SDS-PAGE analysis of the fractions from the main peaks on panel **A**. St, starting material (proteins purified by the MBPtrap chromatography); M, molecular mass markers.

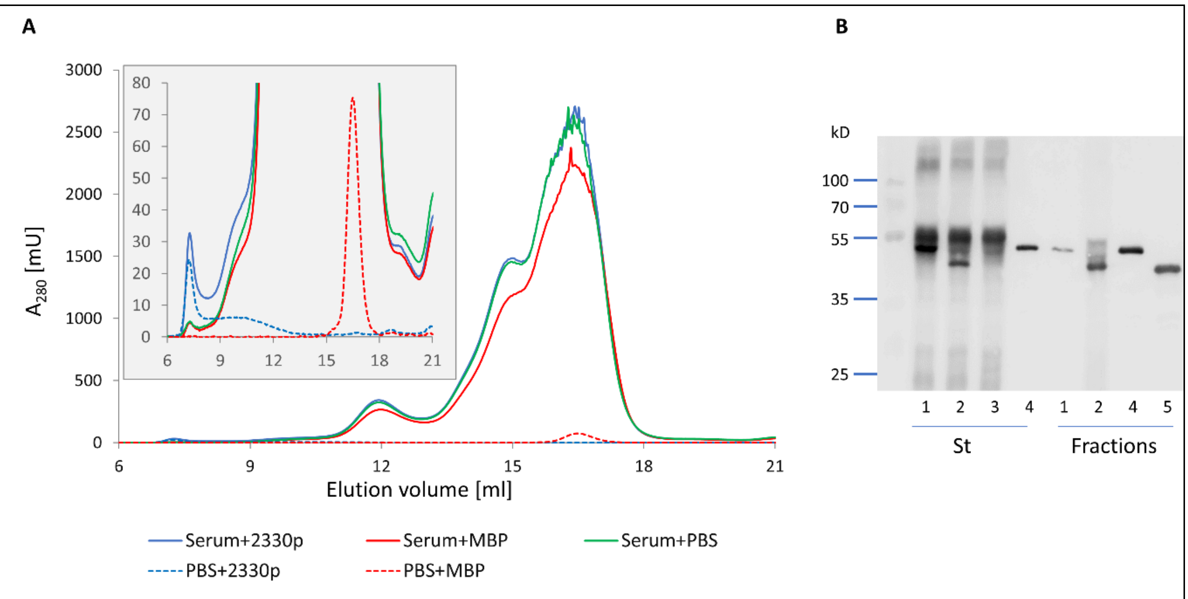

Figure S9. Stability of 2330p oligomer in mouse serum. **A**, samples were prepared as written in **Material and Methods** and run on analytical Superose 6 column. The fractions tested are: (1) mouse serum + 2330p; (2) mouse serum + MBP; (3) mouse serum + PBS; (4) PBS + 2330p; (5) PBS + MBP. An insert represents a magnified view of the chromatogram with the special emphasis on 2330p- and MBP-containing elution volume fractions. X-axis represents elution volume in ml, while Y-axis shows optical density of solution at 280 nm. **B**, starting material ("St") and selected column chromatography fractions ("Fractions", see the legend to panel **A**) were 1/25- and 1/5-diluted respectively and tested in Western blotting with the anti-MBP antibody. Positions of molecular mass markers are shown on the left in kD.

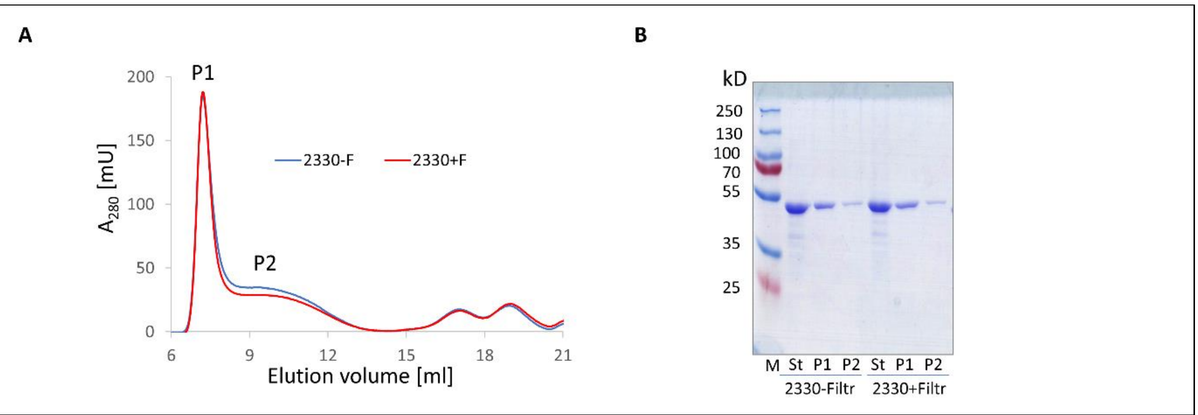

Figure S10. Membrane filtration (0,2 µm pore size) analysis of 2330pept-containing chimeric protein. **A**, analytical gel-chromatography of filtered (2330+F) or not-filtered (2330-F) chimeric 2330p on Superose 6. X-axis represents elution volume in ml, while Y-axis shows optical density of solution at 280 nm. **B**, SDS-PAGE analysis of the fractions from the main peaks on panel A. St, starting material (proteins purified by the MBPtrap chromatography); M, molecular mass markers.

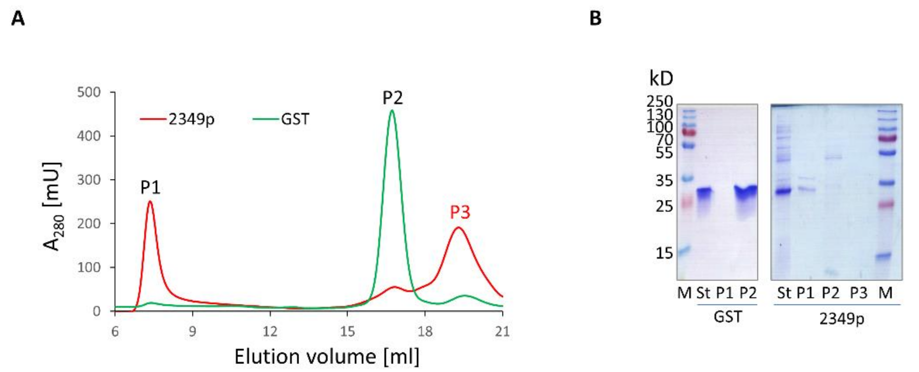

Figure S11. Biochemical analysis of chimeric proteins. **A**, analytical gel-chromatography on Superose 6. Protein 2349p represents GST fused to 2330pept. X-axis represents elution volume in ml, while Y-axis shows optical density of solution at 280 nm. **B**, SDS-PAGE analysis of the fractions from the main peaks on panel A. St, starting material (proteins purified by the MBPtrap chromatography); M, molecular mass markers.

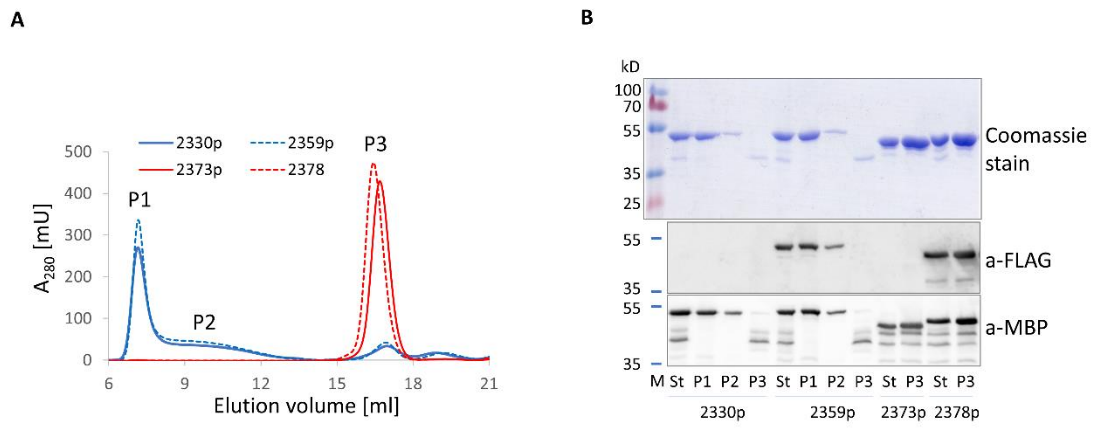

Figure S12. Biochemical analysis of chimeric proteins. **A**, analytical gel-chromatography on Superose 6. Proteins 2359p and 2378p are FLAG-tagged 2330p and 2373p respectively. X-axis represents elution volume in ml, while Y-axis shows optical density of solution at 280 nm. **B**, SDS-PAGE and Western-blot analysis with the anti-FLAG and anti-MBP sera of the fractions from the main peaks on panel A. St, starting material (proteins purified by the MBPtrap chromatography); M, molecular mass markers.

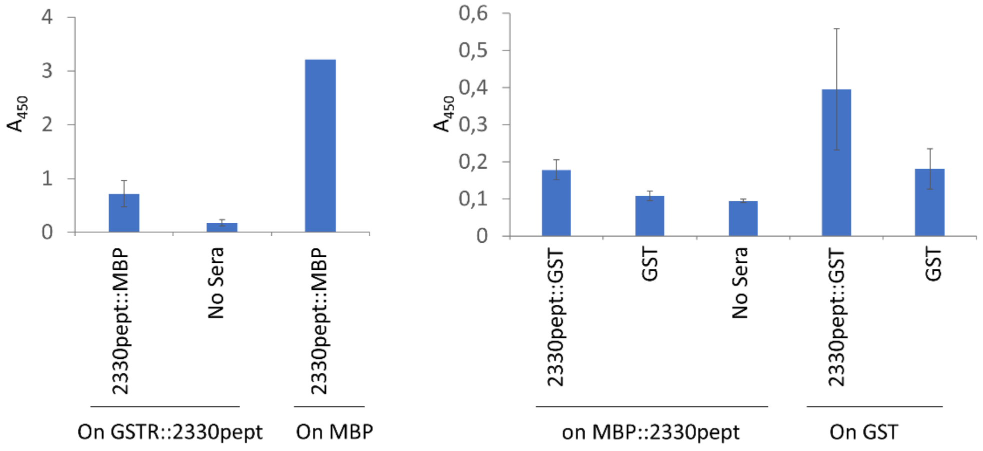

Figure S13. Enzyme-linked immunosorbent assay of mice sera. The animals were immunized with the following proteins: MBP::2330pept (2330p), GST and GST::2330pept (2349p). The sera were tested on plates sensitized with MBP::2330pept ("on MBP::2330pept"), MBP ("on MBP"), GST::2330pept ("on GST::2330pept") or GST ("on GST"). Data are shown as means of triplicates and standard deviations. Y-axis represent optical density (absorbance) measured at 450 nm.

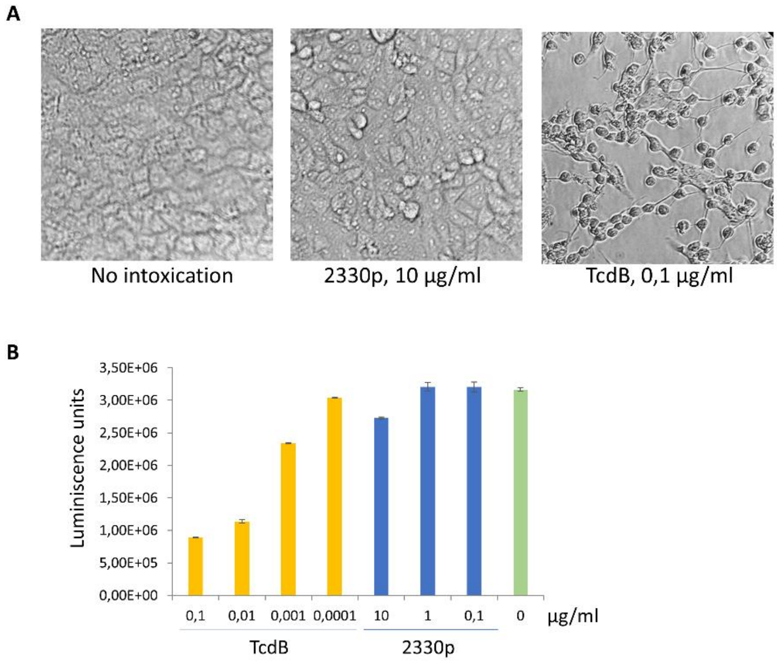

Figure S14. Cytotoxicity of 2330p. **A**, microscopic pictures of Vero cells intoxicated as described in **Materials and Methods** and incubated for 24h. Non-intoxicated cells and cells treated with *Clostridium difficile* toxin TcdB were used as negative and positive controls correspondingly. **B**, cell viability as measured by the CellTiter-Glo® Luminescent Cell Viability Assay. The kit measures ATP levels, which is indicative of metabolic activity and hence viability of the cells.

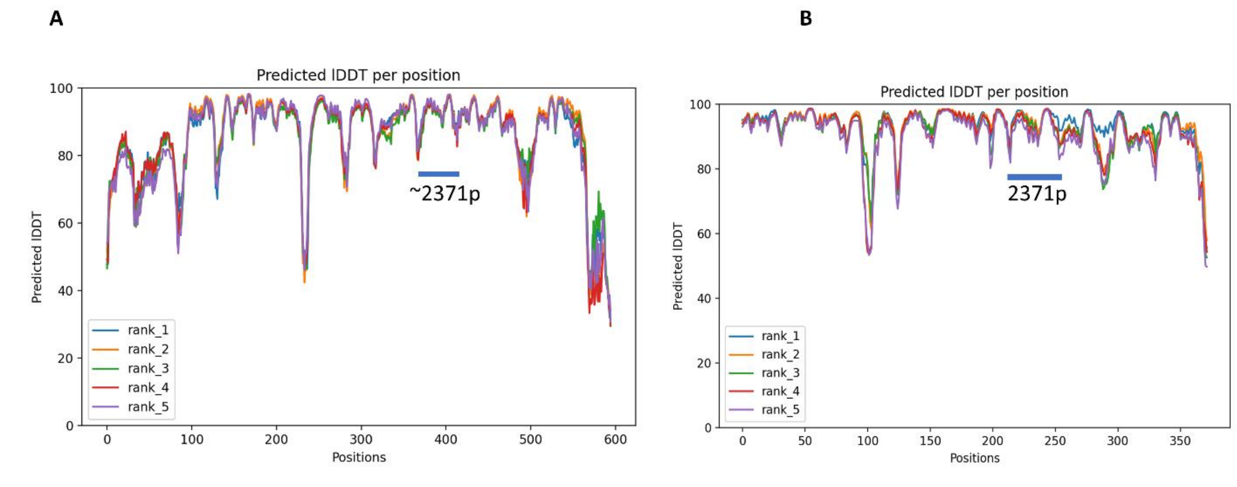

Figure S15. Statistical estimation of AlphaPhold2 modelling quality. **A**, LDDT showing confidence of the predicted structure for VirD4. **B**, LDDT showing confidence of the predicted structure for VirB4. Regions with pLDDT > 90 are expected to be modelled to high accuracy. These should be suitable for any application that benefits from high accuracy (e.g., characterizing binding sites). Regions with pLDDT between 70 and 90 are expected to be modelled well (a generally good backbone prediction). Amino acid residue sequence of 2371pept is shown by the horizontal line.
