# Supplementary material for: Oligomerization and Adjuvant Activity of Peptides Derived from the VirB4-like ATPase of Clostridioides difficile
Source: Biomolecules. 2023 Jun 18;13(6):1012. doi: 10.3390/biom13061012 (PMC10296573; doi:10.3390/biom13061012)
Supplement: Supplementary file 1 [file biomolecules-13-01012-s001.zip › Oligomerization_BelyiY_SuppleTables_2023-05-21.pdf]

Table S1. Primers used for the construction of expression plasmids encoding the VirB4 protein fragments.

NN	Resulting construct	Primers, sence/antisence sequences, RE site	Matrix DNA
1	p2321	#1736, GAACC*CCATGG*ATATAAACCTTAATTAC, NcoI<br>#17, GCTAGTTATTGCTCAGCGG, HindIII	p1360
2	p2322	#1737, CAA*CCATGG*CTATCTTGGGCGAC, NcoI<br>#17, GCTAGTTATTGCTCAGCGG, HindIII	p1360
3	p2323	#1738, GTTAG*CCATGG*TCATTGTGCAAGA, NcoI<br>#17, GCTAGTTATTGCTCAGCGG, HindIII	p1360
4	p2324	#1739, CTACT*CCATGG*ACGAGTTTCACTTG, NcoI<br>#17, GCTAGTTATTGCTCAGCGG, HindIII	p1360
5	p2326	#1740, GATTTATAACAA*AGATCT*GCTGCCG, BglII<br>#1741, GTAAAGCTC*AAGCTT*ATTTCGGGAAG, HindIII	p2323
6	p2327	#1740, GATTTATAACAA*AGATCT*GCTGCCG, BglII<br>#1742, CTTCATCTGCT*AAGCTT*AAATATTAAG, HindIII	p2323
7	p2328	#1740, GATTTATAACAA*AGATCT*GCTGCCG, BglII<br>#1743, TCGCCCT*AAGCTT*AATTGAGCATAAG, HindIII	p2323
8	p2329	#1740, GATTTATAACAA*AGATCT*GCTGCCG, BglII<br>#1744, TGTTCTCAAAGA*AAGCTT*ACACCTC, HindIII	p2323
9	p2330	#1740, GATTTATAACAA*AGATCT*GCTGCCG, BglII<br>#1745, CCCATT*AAGCTT*AACGCTTCCAAAT, HindIII	p2323
10	p2331	#1740, GATTTATAACAA*AGATCT*GCTGCCG, BglII<br>#1746, TCCA*AAGCTT*AGCGGGTGGATTTC, HindIII	p2323
11	p2335	#1740, GATTTATAACAA*AGATCT*GCTGCCG, BglII<br>#1745, CCCATTAAGCTTAACGCTTCCAAAT, HindIII	p2324
12	p2340	#1740, GATTTATAACAA*AGATCT*GCTGCCG, BglII<br>#1761, CAATGAGCA*AAGCTT*ACTTTTTAAGC, HindIII	p2322
13	p2341	#1740, GATTTATAACAA*AGATCT*GCTGCCG, BglII<br>#1762, GCTGCTTTA*AAGCTT*AGCAGACAAG, HindIII	p2322
14	p2342	#1740, GATTTATAACAA*AGATCT*GCTGCCG, BglII<br>#1763, CGTTAAGGC*AAGCTT*AAACATACAG, HindIII	p2322
15	p2343	#1740, GATTTATAACAA*AGATCT*GCTGCCG, BglII<br>#1764, CGGCT*AAGCTT*ACAGCTCGTCGTA, HindIII	p2322
16	p2344	#16, AATACGACTCACTATAGG, NcoI<br>#1768, GGCGTTC*AAGCTT*TACTAAAGGGC, HindIII	p994
17	p2346	#1770, GATTT*CCATGG*GTTTCCGTAAATG, NcoI<br>#1771, TCAGGC*GCTCTTCCGCTT*CCTCGCTCACTG, SapI	p2323
18	p2347	#1740, GATTTATAACAA*AGATCT*GCTGCCG, BglII<br>#1772, CAAGA*AAGCTT*ATTTTGCCGGGTCG, HindIII	p2321
19	p2349	#1773, GAAGGATTTCACAT*GGATCC*ATGGT, BamHI<br>#1774, GGTTATTGTCT*CTCGAG*CGGATAC, XhoI	p2330
20	p2350	#1776, GCAAGCCGCG*CCATGG*AAAATATC, NcoI<br>#1771, TCAGGC*GCTCTTCCGCTT*CCTCGCTCACTG, SapI	p2346
21	p2351	#1777, CTTGCAAAGCAGC*CCATGG*TTTCCC, NcoI<br>#1771, TCAGGC*GCTCTTCCGCTT*CCTCGCTCACTG, SapI	p2346
22	p2352	#1778, GACCGCTTCC*CCATGG*ATACCGAG. NcoI<br>#1771, TCAGGC*GCTCTTCCGCTT*CCTCGCTCACTG, SapI	p2346
23	p2353	#16, TAATACGACTCACTATAGG, NcoI<br>#1779, GTTTA*AAGCTT*AAGGGTTCACATAC, HindIII	p1360
24	p2354	#1740, GATTTATAACAA*AGATCT*GCTGCCG, BglII<br>#1741, GTAAAGCTC*AAGCTT*ATTTCGGGAAG, HindIII	p2346
25	p2355	#1740, GATTTATAACAA*AGATCT*GCTGCCG, BglII<br>#1742, CTTCATCTGCT*AAGCTT*AAATATTAAG, HindIII	p2346
26	p2356	#1740, GATTTATAACAA*AGATCT*GCTGCCG, BglII<br>#1743, TCGCCCT*AAGCTT*AATTGAGCATAAG, HindIII	p2346

27	p2357	#1740, GATTTATAACAA*AGATCT*GCTGCCG, BglII<br>#1744, TGTTCTCAAAGA*AAGCTT*ACACCTC, HindIII	p2346
28	p2359	#1740, GATTTATAACAA*AGATCT*GCTGCCG, BglII<br>#1782, TATAT*AAGCTT*ACTTATCGTCGTCATCCTTGTAATCCATCGATCGCT TCCAAATCTCCACGCTGT, HindIII	p2330
29	p2360	#1780, TATG*GGATCCGTCGACCTGCAGGAATTCGGTAC*<br>#1781, CAT*GGTACCGAATTCCTGCAGGTCGACGGATCC*CA	Self-annealing
30	p2361, p2363	#154, TACGGTG*GGATCC*CTATATAAGCAGAGC, BamHI<br>#155, TTGTGAAATTTGTGATGCTATTGCTT	pEGFP-C
31	p2370	#1740, GATTTATAACAA*AGATCT*GCTGCCG, BglII<br>#1786, CTCTTTTA*AAGCTT*AGTGAAACTCGTC, HindIII	p2330
32	p2371	#1787, CAAGAC*CATATG*TGGAACAGGGTAAC, NdeI<br>#1771, TCAGGC*GCTCTTCCGCTT*CCTCGCTCACTG, SapI	p2330
33	p2373	#1787, CAAGAC*CATATG*TGGAACAGGGTAAC, NdeI<br>#1771, TCAGGC*GCTCTTCCGCTT*CCTCGCTCACTG, SapI	p2370
34	p2374	#1740, GATTTATAACAA*AGATCT*GCTGCCG, BglII<br>#1790, AAAC*AAGCTT*AAATCTCCACGCTGTAAGC, HindIII	p2371
35	p2375	#1740, GATTTATAACAA*AGATCT*GCTGCCG, BglII<br>#1791, AAAC*AAGCTT*AGTAAGCTGCCGTCTGTTC, HindIII	p2371
36	p2376	#1740, GATTTATAACAA*AGATCT*GCTGCCG, BglII<br>#1792, AAAC*AAGCTT*ACTGTTCCTCTTTTAAGAGC, HindIII	p2371
37	p2377	#1740, GATTTATAACAA*AGATCT*GCTGCCG, BglII<br>#1793, AAAC*AAGCTT*ATAAGAGCAAGTGAAACTCG, HindIII	p2371
38	p2378	#1740, GATTTATAACAA*AGATCT*GCTGCCG, BglII<br>#1788, AAAC*AAGCTT*ACTTATCGTCGTCATCCTTGTAATCCATGGGGTGAAAC TCGTCCATATA, HindIII	p2373

Table S2. Selective properties of the VirB4-derived peptides. Peptides with high, intermediate, and low oligomerization activities are shown in red, blue, and green respectively. Data on the peptide 2330pept are shown in red bold font.

NN	Peptide ID	Length (aa)	pI	Hydrophobic aa (AILFWV), %
1	1361pept	372	5,66	35,8
2	2321pept	279	5,44	36,9
3	2322pept	215	6,4	37,2
4	2323pept	152	6,19	35,5
5	2324pept	127	5,19	37,0
6	2326pept	134	8,15	37,3
7	2327pept	101	8,22	37,6
8	2328pept	86	6,8	37,2
9	2329pept	72	9,45	36,1
10	**2330pept**	**48**	**8,31**	**33,3**
11	2331pept	23	10,9	30,4
12	2335pept	23	4,96	39,1
13	2340pept	61	6,77	41,0
14	2341pept	49	4,75	42,9
15	2342pept	28	4,08	50
16	2343pept	11	3.49	43
17	2346pept	104	5,33	35,5
18	2347pept	62	4,43	37,1
19	2350pept	80	4,75	35
20	2351pept	51	5,09	31,4
21	2352pept	19	4.41	19.5
22	2353pept	93	7,97	32,3
23	2354pept	86	6,79	39,5
24	2355pept	53	6,3	41,5
25	2356pept	38	4,94	42,1
26	2357pept	24	9,99	41,7
27	2370pept	30	8,44	26,7
28	2371pept	40	9,23	32,5
29	2373pept	22	9,52	22,7
30	2374pept	38	6,78	31,6
31	2375pept	34	8,34	29,4
32	2376pept	30	8,39	26,7
33	2377pept	26	9,52	30,8
34	MBP	426	5,82	34,0
